# Intracellular Presence of *Helicobacter pylori* and Its Virulence-Associated Genotypes within the Vaginal Yeast of Term Pregnant Women

**DOI:** 10.3390/microorganisms9010131

**Published:** 2021-01-08

**Authors:** Kimberly Sánchez-Alonzo, Lillian Matamala-Valdés, Cristian Parra-Sepúlveda, Humberto Bernasconi, Víctor L. Campos, Carlos T. Smith, Katia Sáez, Apolinaria García-Cancino

**Affiliations:** 1 Laboratory of Bacterial Pathogenicity, Department of Microbiology, Faculty of Biological Sciences, Universidad de Concepcion, Concepción 4070386, Chile; kimsanchez@udec.cl (K.S.-A.); cparras@udec.cl (C.P.-S.); csmith@udec.cl (C.T.S.); 2 Department of Obstetrics, Faculty of Medicine, Universidad de Concepción, Concepción 4070386, Chile; lmatamala@udec.cl; 3 Laboratorio Pasteur, Concepción 4030000, Chile; hbernasconi@lpasteur.cl; 4 Laboratory of Environmental Microbiology, Department of Microbiology, Faculty of Biological Sciences, Universidad de Concepcion, Concepción 4070386, Chile; vcampos@udec.cl; 5 Department of Statistics, Faculty of Physical and Mathematical Sciences, Universidad de Concepcion, Concepción 4070386, Chile; ksaez@udec.cl

**Keywords:** intracellular *H. pylori*, genotypes, *C. albicans*, transmission, vaginal discharge

## Abstract

Background: *Helicobacter pylori* transmission routes are not entirely elucidated. Since yeasts are postulated to transmit this pathogen, this study aimed to detect and genotype intracellular *H. pylori* harbored within vaginal yeast cells. Methods: A questionnaire was used to determine risk factors of *H. pylori* infection. Samples were seeded on Sabouraud Dextrose Agar and horse blood-supplemented Columbia agar. Isolated yeasts were identified using and observed by optical microscopy searching for intra-yeast *H. pylori*. Total yeast DNA, from one random sample, was extracted to search for *H. pylori* virulence genes by PCR and bacterial identification by sequencing. Results: 43% of samples contained yeasts, mainly *Candida albicans* (91%). Microscopy detected bacteria such as bodies and anti-*H. pylori* antibodies binding particles in 50% of the isolated yeasts. Total DNA extracted showed that 50% of the isolated yeasts were positive for *H. pylori* 16S rDNA and the sequence showed 99.8% similarity with *H. pylori.* In total, 32% of *H. pylori* DNA positive samples were *cagA+ vacAs1a vacAm1 dupA−*. No relationship was observed between possible *H. pylori* infection risk factors and vaginal yeasts harboring this bacterium. Conclusion: *H. pylori* having virulent genotypes were detected within vaginal yeasts constituting a risk for vertical transmission of this pathogen.

## 1. Introduction

*H. pylori*, a Gram-negative bacterium infecting 50% of the world population, possesses multiple virulence factors, such as proteins CagA, VacA and DupA [1,2,3,4], making it a primary pathogen associated to various gastric pathologies including peptic ulcer, mucosa-associated gastric lymphoma and gastric cancer [5]. This pathogen has also been associated to extra-gastric pathologies, such as hyperemesis gravidarum, ischemic stroke, Alzheimer’s disease, rosacea and iron deficiency anemia, Non-alcoholic fatty liver disease and open-angle glaucoma [6,7,8,9,10,11].

The way in which *H. pylori* persists in the environment, as well as the factors facilitating its entry into human gastric epithelial cells and its transmission mechanism from person to person, remains unknown. The chronic nature of the infection produced by *H. pylori*, the high prevalence of asymptomatic infected individuals, the difficulties to culture *H. pylori* and the impossibility to culture it from some environments where it has been detected by molecular techniques have hindered the knowledge on how this bacterium is able to reach the human stomach [12,13].

On the other hand, intracellular life of prokaryotes within eukaryotic cells is considered a major evolutionary phenomenon which led to the adaptation of prokaryotes to a wide range of environmental niches. However, largely due to the inability to culture these intracellular bacteria, the details of this type of relationship have not been yet elucidated [14,15].

*H. pylori* resides predominantly on the surface of gastric epithelial cells and in the overlying mucus and only few *H. pylori* cells enter into host’s epithelial and immune cells [16,17]. Microscopic observations have detected the presence of *H. pylori* within vacuoles of epithelial cells, macrophages and dendritic cells [17,18,19,20]. Thus, *H. pylori* has been described as a facultative intracellular bacterium which has evolved to make use of vacuoles of eukaryotic cells as a protective niche, allowing it to multiply and persist there during a long time [13,15,21]. Amoebae and yeasts have been reported as other eukaryotic cells whose vacuoles are used by *H. pylori* as a protective niche. Yeasts are highly sophisticated organisms with a remarkable ability to adapt to environmental stress and to the antimicrobial activity of the host’s immune system [21,22,23]. Yeasts of the genus *Candida* are commonly found in the skin and mucous membranes of humans, including the gastric mucosa [23,24,25], while *H. pylori* is found in the human stomach and duodenum. Therefore, both microorganisms are well adapted to the gastric environment [23,26,27]. The interaction of these microorganisms may have started a long time ago, leading to the internalizing of *H. pylori* into vacuoles of yeasts as a prior adaptation to invade and persist inside human immune and epithelial cells.

In Chile, the infection rate by *H. pylori* is high, starts at young ages and it has a strong association with gastric cancer [28].There is solid evidence to support the view that the principal reservoir of *H. pylori* are other humans and that the principal mode of transmission is from person to person within the family group [29,30,31]. However, the exact timing of bacterial transmission is unknown. Therefore, this work was aimed to detect, identify and genotyping intracellular *H. pylori* within yeasts of vaginal origin in term pregnant women and also to correlate this association with possible *H. pylori* infection risk factors.

## 2. Materials and Methods

### 2.1. Ethical Considerations

This study was approved on July 2015 by the Scientific Ethical Committee of the Concepción, Health Service, Chile under the code 06/15–22. The purpose of this study and the sampling procedure were duly informed to the prospective participants. In addition, women participating in this study were notified that their participation was voluntary and that they could withdraw at any stage of it. Written informed consent was obtained from all participants.

### 2.2. Patients

Samples were collected during the final stage of pregnancy from the posterior fornix of 102 pregnant women being controlled at three state Family Health Centres (O’Higgins, Tucapel and Dr. Víctor Manuel Fernández) at Concepción, Chile. Since patients were randomly selected and it was unknown if they were infected with *H. pylori* or not, a questionnaire was prepared to determine if women had risk factors or symptoms of the infection with this pathogen. The questionnaire included the following questions: Have you had previous spontaneous abortions? Do you or the father of your child have received treatment against *H. pylori*? During pregnancy, have you been diagnosed as having *H. pylori*? During pregnancy, have you received treatment against vaginal candidiasis? Do you suffer some of the following pathologies: hyperemesis gravidarum, pre-eclampsia or iron-deficiency anemia? Which of the following types of sex do you practice: vaginal sex, oral sex, anal sex.

### 2.3. Sample Processing

Samples were obtained, using a cotton swab, from the posterior fornix and transferred into tubes containing Stuart transportation medium (Deltalab, Barcelona, Spain) to be transported to the Bacterial Pathogenicity Laboratory, University of Concepción, for their analysis. Each sample was inoculated onto plates containing Sabouraud Dextrose Agar (SDA) (Merck, Darmstadt, Germany) supplemented with chloramphenicol (OXOID, Basingstoke, UK), following the directions of the manufacturer. The purpose of adding chloramphenicol was to eliminate extracellular bacterial contamination, including *H. pylori* sensitive to this antibiotic [32,33]. Samples were disseminated covering all the surface of the plate using a swab and incubated at 37 °C for 24 h under aerobic conditions. Following incubation, all colonies on the chloramphenicol supplemented SDA plates were Gram stained to verify the presence of yeast cells and to confirm the absence of extracellular bacteria. In order to decrease the risk of contamination, each primary culture was reseeded four times in plates containing the same medium than the primary culture (chloramphenicol supplemented SDA) and incubated under the same conditions. Then, the yeasts isolated from the vaginal samples were identified by means of the CHROMagar *Candida* medium (Difco, Wokingham, UK) and the API *Candida* identification system (BIOMÉRIEUX, Craponne, France) following the instructions of the manufacturers. In addition, a Gram staining was performed to each reseeded colony to confirm the purity of the culture. Each sample was also inoculated in Columbia agar (OXOID, Basingstoke, UK) supplemented with DENT (OXOID, Basingstoke, UK) following the directions of the manufacturer to discard that *H. pylori* could have been present in the vagina outside of yeasts. These samples were incubated under microaerobic conditions (10% CO_2_) at 37 °C for 5 days.

### 2.4. Search for Bacteria-Like Bodies (BLBs) within Vaginal Yeasts

From all the pure cultures grown four times on plates containing SDA plus chloramphenicol, inocula were obtained from randomly chosen colonies and placed on slides containing 20 µL of 0.9% saline solution. Then, coverslips were placed on the samples and observed using an optical microscope (Leica, Wetzlar, Germany) equipped with a 100× oil immersion objective lens and camera to search for the presence of BLBs.

### 2.5. Amplification of H. pylori Specific Genes from the DNA of Yeasts

DNA from vaginal yeasts and from control strains [*H. pylori* ATCC (American Type Culture Collection) 43504 as positive control and *C. albicas* ATCC 90028 as negative control] was extracted by means of the UltraClean Microbial DNA Isolation kit (M.O. BIO, Carlsbad, CA, USA) following the instructions of the manufacturer. *H. pylori* genes were amplified using the Sapphire-Amp Fast PCR Master Mix kit (TAKARA BIO INC, Otsu, Japan). For each assay, 12.5 μL of Master Mix, 1 μL of primers specific for *H. pylori* genes (see Table 1), 1.5 μL of the DNA of the sample and 5.5 μL of PCR-grade water were added to obtain a final volume of 25 μL of PCR mixture.

PCR conditions were as follows: initial denaturation at 94 °C/1 min, denaturation temperature 98 °C/5 s, hybridization temperature for each primer as indicated in Table 1 and extension at 72 °C/40 s. Thirty cycles for each PCR reaction were done using an Eppendorf thermocycler (Hauppauge, New York, NY, USA). Amplification of the genes was confirmed after 2% agarose gel electrophoresis (Lonza, Walkersville, MD, USA) plus 1.6 μL RedGel (Biotium, San Francisco, CA, USA) run at 80 V for 90 min and the gels were visualized under UV light using an Enduro model transilluminator (Labnet, Edison, NJ, USA).

### 2.6. Detection of H. pylori by Immunofluorescence

In order to make sure that all possible extracellular bacteria were eliminated, yeasts isolated from vaginal discharges were cultured four times in chloramphenicol supplemented SDA. The fourth sub-culture and controls (negative control *Candida albicans* ATCC 90028, positive control *H. pylori* 43504) were independently transferred to Eppendorf tubes containing 1 mL phosphate-buffered saline (PBS) 1× pH 7.4 and adjusted to a turbidity similar to the pattern of a number 2 tube of the McFarland scale. Two hundred μL of each sample were added to a well of a 96-wells plate and then 1 μL of 5 mg mL^−1^ fluorescein isothiocyanate (FITC)-labeled anti-*H. pylori* polyclonal IgG antibodies (Abcam, Cambrige, UK) was added to each well and incubated for 1 h at room temperature in the dark. Then, each sample was independently transferred to 1.5 mL Eppendorf tubes and washed twice to eliminate unbound antibodies. This was accomplished adding 500 mL PBS 1×, vortexing for 5 s and centrifuging at 10,000 rpm for 2 min. A total of 10 μL of each sample were added to a slide and observed at a wavelength of 528 using a LSM780 NLO spectral confocal microscope (ZEISS, Berlin, Germany) achieving fluorescence with a laser excitation at 488 nm and emission between 490–560 nm. Images were obtained using differential interference contrast (DIC) microscopy (transmitted light images).

### 2.7. Amplification and Sequencing of 16S rDNA of H. pylori

From the samples confirmed, by microscopy and PCR amplification, to be positive for intracellular *H. pylori*, one sample was randomly selected, and total DNA was extracted from yeasts using the UltraClean Microbial DNA Isolation kit (M.O. BIO, Carlsbad, CA, USA) following the instructions of the manufacturer. The 16S rDNA was amplified by PCR using universal bacterial primers 8F-(5′-AGTTTGATCCTGGCTCAG-3′), 1492R (5′-ACCTTGTTACGACTT-3′). The amplified fragments were purified and sequenced by Macrogen Inc. (Seoul, Korea). To determine the taxonomic allocation of the fragments sequenced, their phylogenetic affiliation was analysed comparing the 16S rRNA gene sequence. Sequences were revised and corrected using Sequencer v4.7 software (Gene Codes Corp, Ann Arbo, MI, USA). The sequences were added to the updated and prealigned 16S rRNA gene database Silva (http://www.arb-silva.de/projects/living-tree/), compiling all sequences of all type strains for which an entry of high quality was found [34]. The sequences were aligned using the ARB software package (http://www.arb-home.de) [35] and manually improved. The tree reconstruction was performed using the neighbor-joining algorithm implemented in the ARB software package. The sequences were submitted to GenBank with the following access number MT477178.

### 2.8. Statistical Analysis

Results of qualitative variables were incorporated into a database and processed using SPSS 24.0 software (IBM Company, Armonk, NY, USA). Levels of the categorical variables were expressed by their frequencies and percentages. The chi-square test was used to determine the relationship between categorical variables. A significance level of *p* < 0.05 was used.

## 3. Results

### 3.1. Patients

The age of women who accepted to participate in this study ranged between 14 and 44 years old (Table 2).

The Family Health Centre. “Dr. Víctor Manuel Fernández” contributed the largest percentage of samples positive for *H. pylori* containing yeasts (75%) (Table 3).

Regarding the answers provided by the patients to the survey, none of the women reported spontaneous abortions nor diagnosis of *H. pylori* infection, hyperemesis gravidarum, pre-eclampsia or iron-deficiency anemia. Regarding the diagnosis of vaginal candidiasis during pregnancy, 14% of the women participating in the study reported to had been diagnosed and treated against this infection; nevertheless, none of them reported to have received treatment for mycotic vulvovaginitis during the last month or being treated with an antimycotic at the moment of sampling.

### 3.2. Isolation and Identification of Yeasts and H. pylori

From the 102 samples of vaginal discharge obtained from term pregnant women at the three health centers, 44 of them were positive for yeasts. According to the growth in CHROMagar and the identification by API, the 44 samples positive for yeast included *C. albicans* (40 isolates, 91%), *C. glabrata* (3 isolates, 7%) and *C. tropicalis* (1 isolate, 2%). All samples of vaginal discharge cultured in Columbia agar were negative for the growth of *H. pylori*.

### 3.3. Detection of BLBs and H. pylori by Optical Microscopy

**Figure 1:**
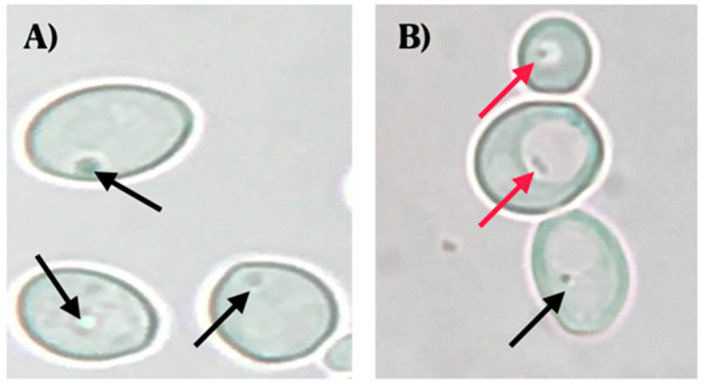
Wet mounts (objective lens magnification 100×) of yeast cells obtained from colonies cultured on chloramphenicol-supplemented Sabouraud Dextrose Agar. (**A**) *C. albicans* ATCC 90028 strain (negative control) (objective lens magnification 100×). (**B**) *C. albicans* isolated from a vaginal discharge sample of a term pregnant woman showing the presence of bacteria-like bodies (BLBs) within vacuoles of yeast. Red arrows indicate BLBs, while black arrows indicate nuclei of yeast cells. Micrograph B is a representative image of one of the triplicates of one of the vaginal discharge samples positive for the presence of intra-yeast bacteria-like bodies.

Observations using optical microscopy showed the presence of BLBs in 22 of the 44 vaginal discharges positive for yeasts. The movement of the BLBs was restricted to the vacuolar space of yeasts (Figure 1).

**Figure 2:**
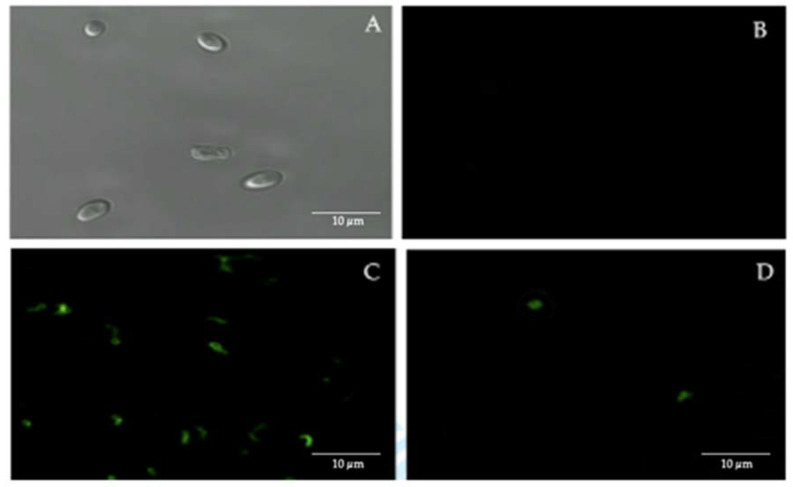
Immunofluorescence assay using fluorescein isothiocyanate (FITC)-labeled anti-*H. pylori* IgG polyclonal antibodies. (**A**) bright field microscopy of *C. albicans* ATCC 90028 strain (negative control). (**B**) *C. albicans* ATCC 90028 strain (negative control) showing the absence of fluorescence. (**C**) *H. pylori* ATCC 43504 strain (positive control) showing fluorescence. (**D**) fluorescent intracellular *H. pylori* within yeast cells isolated from the vaginal discharge sample, cultured in Sabouraud agar supplemented with chloramphenicol, of a term pregnant woman. Micrograph D is a representative image of one of the triplicates of one of the vaginal discharge samples positive for the presence of intra-yeast *H. pylori.*

Confocal fluorescence microscopy, using FITC-labeled anti-*H. pylori* antibodies, showed the presence of intracellular *H. pylori* in 50% of the 44 vaginal discharge samples of pregnant women positive for yeasts (Figure 2).

**Figure 3:**
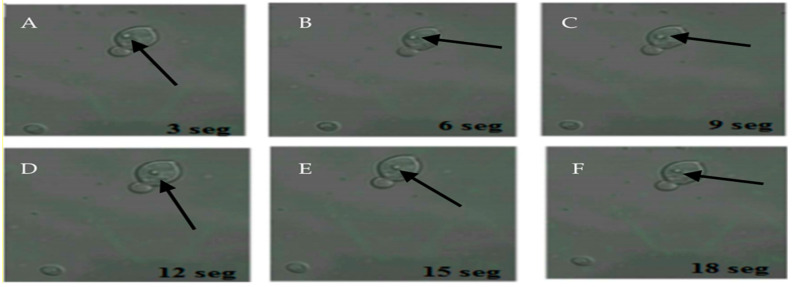
Movement of *H. pylori* within yeasts of vaginal discharge origin. Confocal microscopy images taken 3 s apart using a Zeiss LSM780 NLO confocal microscope. (**A**–**F**) show the movement of *H. pylori* (arrows) inside the vacuole of a yeast cell. Light green color observed in the background represent remnants of fluorescein isothiocyanate (FITC). This figure is representative of images of one of the triplicates of one of the vaginal discharge samples positive for the presence of intra-yeast *H. pylori.*

The 22 samples positive for FITC-labeled antibodies were the same samples positive for the presence of BLBs. Therefore, the term BLBs will be hereafter replaced by *H. pylori* throughout the text. In addition, it was possible to observe bacteria moving within the yeasts (Figure 3).

### 3.4. Relationship of Intracellular H. pylori with Age or Sexual Practices of Participants

Among the yeasts isolated, 33% of them were obtained from pregnant women within the 35–44 years age range. No significant differences in the frequency of intracellular *H. pylori* harboring yeasts were found among the different age groups (Table 2). Regarding a possible relationship between sexual practices and the percentage of women positive for *H. pylori* harboring yeasts, after comparing the distribution and frequency percentages the *p* value was above the significance limit, indicating that, at least in this study, no relationship was found between the percentage of women positive for intracellular *H. pylori* harboring yeasts and sexual practices (Table 4). Regarding the remaining questions answered by the patients, their answers were no and there was no correlation with the presence or absence of intracellular *H. pylori.*

### 3.5. Amplification of H. pylori Specific Genes in Yeasts

**Figure 4:**
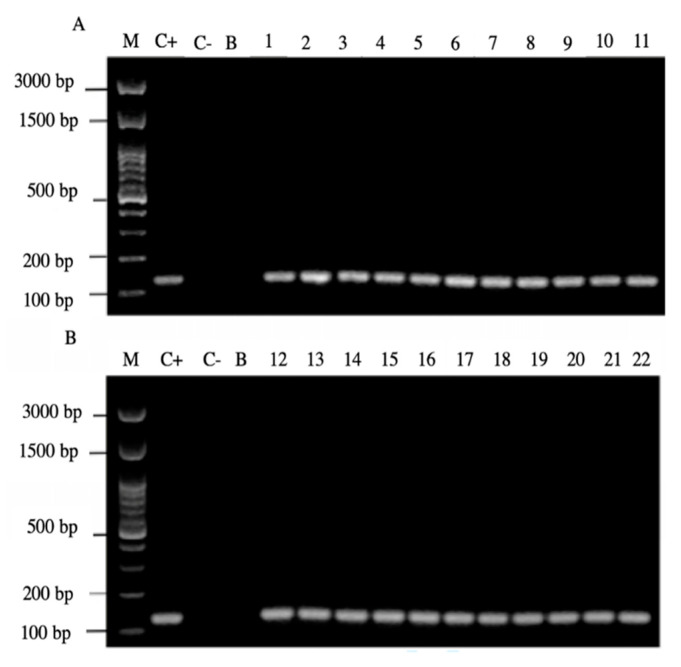
Agarose gel electrophoresis to detect the PCR amplification of the *16S rRNA* gene of *H. pylori* (expected size 100 pb) from the DNA of yeast cells from vaginal discharges of term pregnant women. Lane M: molecular weight markers. Lane C+: DNA extracted from *H. pylori* ATCC 43504 strain (positive control). Lane C−: DNA extracted from *C. albicans* ATCC 90028 strain (negative control). Lane B: blank (PCR grade water) Lanes 1–11 (**A**) and 12–22 (**B**) DNA extracted from yeasts harboring bacteria-like bodies obtained from the vaginal discharge of term pregnant women.

Of the 44 positive samples for yeasts, 22 of them (50%) were positive for the amplification of the *H. pylori* 16S rRNA gene (Figure 4).

**Figure 5:**
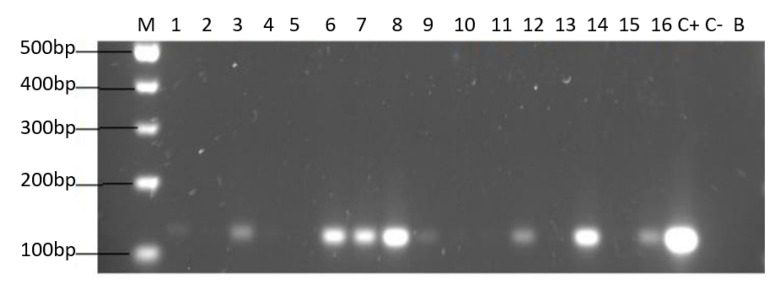
Agarose gel electrophoresis to detect the PCR amplification of the *16S rRNA* gene of *H. pylori* (expected size 100 pb) from the DNA of yeast cells from vaginal discharges of term pregnant women. Lane M: molecular weight markers. Lane C+: DNA extracted from *H. pylori* ATCC 43504 strain (positive control). Lane C−: DNA extracted from *C. albicans* ATCC 90028 strain (negative control). Lane B: blank (PCR grade water) Lanes 2, 4, 5, 10, 11, 13 and 15 correspond to yeasts strains free of bacteria-like bodies and did not amplified the gene. Lanes 1, 3, 6, 7, 8, 9, 12, 14 and 16 correspond to yeasts strains containing bacteria-like bodies and they did amplify the gene.

Only yeast strains harboring BLBs (*H. pylori*) were positive for the amplification of the *H. pylori* 16S rRNA gene (Figure 5).

**Figure 6:**
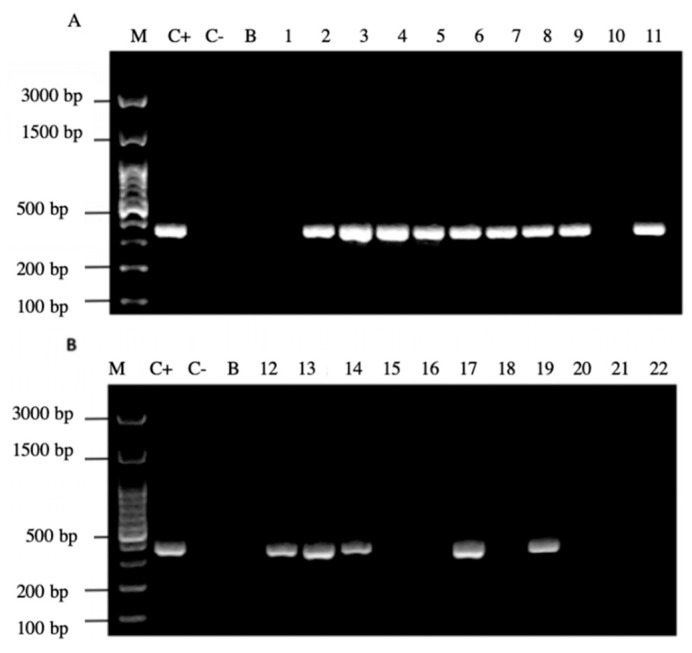
Agarose gel electrophoresis to detect the PCR amplification of the *cagA* gene of *H. pylori* (expected size 349 pb) from the DNA of yeast cells from vaginal discharges of term pregnant women. Lane M: molecular weight markers. Lane C+: DNA extracted from *H. pylori* ATCC 43504 strain (positive control). Lane C−: DNA extracted from *C. albicans* ATCC 90028 strain (negative control). Lane B: blank (PCR grade water). Lanes 1–11 (**A**) and 12–22 (**B**) DNA extracted from yeasts obtained from the vaginal discharge of term pregnant women.

It is noteworthy to emphasize that samples positive for the amplification of this gene coincided with those samples which were positive for the observation of BLBs and fluorescent intracellular *H. pylori*. Among the 22 samples positive for the *H. pylori* 16S rRNA gene, 14/22 (64%) of them were positive for the *cagA* gene, one of the major virulence markers of *H. pylori* (Figure 6), no sample amplified for the detection of the gene *dupA*.

**Figure 7:**
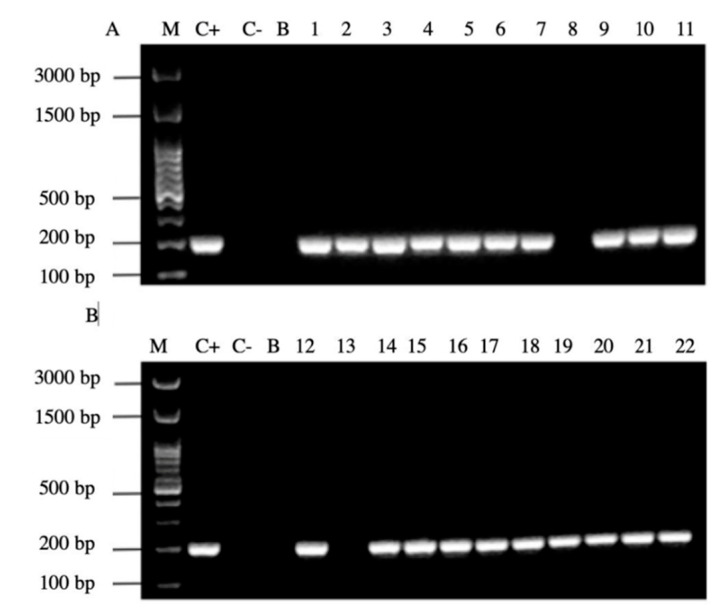
Agarose gel electrophoresis to detect the PCR amplification of the signal region *s1a* of *vacA* gene of *H. pylori* (expected size 190 pb) from the DNA of yeast cells from vaginal discharges of term pregnant women. Lane C+: DNA extracted from *H. pylori* ATCC 43504 strain (positive control). Lane C−: DNA extracted from *C. albicans* ATCC 90028 strain (negative control). Lane B: blank (PCR grade water) Lanes 1–11 (**A**) and 12–22 (**B**) DNA extracted from yeasts obtained from the vaginal discharge of term pregnant women.

In addition, 20/22 (90%) of the PCR products of the samples amplified the signal region *s1a*, coinciding with the positive control *H. pylori* ATCC 43504 strain (Figure 7).

Alleles *vacAs1b* and *vacAs2* were not detected by PCR In relation to the middle region of the gene *vacA*, 9/22 (40%) samples amplified for the region *m1* All profiles obtained are shown in Table 5.

**Figure 8:**
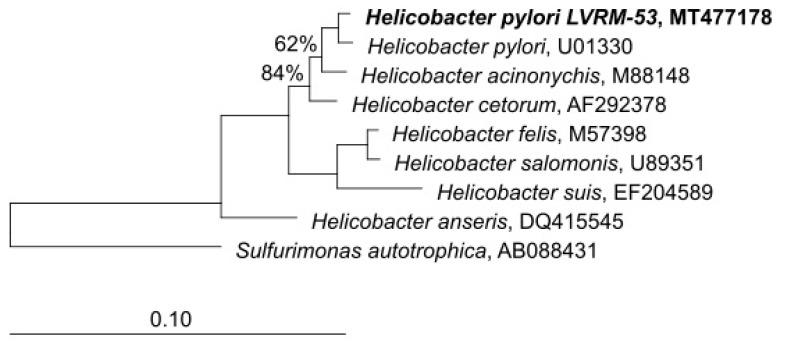
Tree-reconstruction-based on 16S rRNA sequences of LVRM-53 strains, isolated from term pregnant women vaginal yeasts. The tree reconstruction was performed using the neighbor-joining algorithm, implemented in the ARB software package. (GenBank MT477178).

In order to check the taxonomic allocation of the bacterial strains isolated from the yeasts of vaginal origin, we studied the phylogenetic affiliation based on 16S rRNA gene sequence comparisons. The closest GenBank matches for 16S rDNA sequences revealed that the strains showed a high degree of similarity (99.8%) with sequences of *H. pylori* available at the GenBank database. The phylogenetic tree reconstruction performed using the neighbor-joining algorithm, affiliated LVRM-53 (GenBank MT477178) to the genus *Helicobacter sensu stricto* (Figure 8).

## 4. Discussion

Due to its high prevalence and associated pathologies, *H. pylori* is the subject of detailed studies. Nevertheless, the manner in which it disseminates is not yet fully understood [36,37,38,39,40]. So far, *H. pylori* genes have been amplified from different samples of human origin, such as oral plaque, feces, nasal secretions, lacrimal fluid and saliva, the oral cavity being considered one of the main sources of infection [41,42]. Besides having been detected in human samples, this bacterium has also been isolated from fruits and vegetables, processed foods, such as hamburgers and ground meat and from different water sources. Presently, the most accepted transmission routes for this bacterium are oral-oral, gastro-oral and oral-fecal [1,4,12,43,44,45].

In developing countries, *H. pylori* infection occurs early in life, showing the highest infection rates before 10 years of age. Nevertheless, the age of highest susceptibility in infants remains unknown [46], making it necessary to elaborate prevention strategies for children for which it is required to investigate all the possible transmission routes of this bacterium. It is widely accepted that person-to-person transmission occurs, the intra-family transmission being the most frequent one, and the mother, given her close contact with all members of the family, must be considered an important risk factor [12,13]. Besides, there is evidence showing that the vertical transmission can play an important role in the early infection of infants and newborns with *H. pylori*, it being possible to acquire this intracellular pathogen by means of yeasts during the passage through the birth canal [30].

Regarding the results reported in the present study, the largest percentage of the vaginal discharge samples was obtained at the “Dr. Víctor Manuel Fernández” Family Health Centre. This health centre provides health care to a large number of pregnant women residing in the city of Concepción and its surroundings because it also receives women from nearby rural areas. For this same reason, this centre also contributed with a large percentage of the samples positive for yeasts from term pregnant women. Most of pregnant women who participated in the study were in the age range of 21–34 years. This result is in agreement with that reported by the Program of Integral Health of Adolescents and Young People in Chile, indicating that the largest number of pregnancies in this country occurs in this age range [47].

There is evidence that *H. pylori* infection during pregnancy is associated to extra-gastric pathologies, such as hyperemesis gravidarum, pre-eclampsia or iron-deficiency anemia [48,49,50,51].

For this reason, we attempted to determine if the presence of vaginal yeasts harboring *H. pylori* in term pregnant women caused in them the extra-gastric symptomatology associated to this pathogen. Nevertheless, none of the participants of this study reported to suffer any of the above-mentioned pathologies during their pregnancies and stated not to be infected with *H. pylori*. Therefore, the present study was unable to determine that the presence of *H. pylori* harboring intravaginal yeasts was related to the extra-gastric symptomatology caused by this bacterium.

One of the questions we asked ourselves when doing this study was how does intracellular *H. pylori* harbored in yeasts reach the vagina? The literature report evidences that these two microorganisms have been detected in the same locations of the human body, such as mouth and stomach, and both can also be detected in human faeces, implying that both travel through the intestine [52,53,54,55,56]. It is also well known that microorganisms of the intestinal microbiota reach the vagina, ascending because the anatomy of women is such that the vagina and the anus are in close proximity [57]; perhaps this may be the possible answer to our question. On the other hand, since the oral cavity is considered an important source of infection and it is also a habitat for yeasts and *H. pylori*, and since the literature also postulates that *H. pylori* infection could be acquired by sexual practices [58,59], we considered the possibility that sexual practices may also play an important role to allow the arrival of *H. pylori* harbouring yeasts to the vagina. Nevertheless, in accordance with the data from our survey, sexual practices reported by pregnant women have no influence on the presence of vaginal intra-yeast *H. pylori* (anal sex *p =* 0.5159, oral sex *p =* 0.4757).

Regarding the isolation of *H. pylori* from the vaginal discharge samples, all Columbia agar cultures were negative for the growth of this bacterium. These results are consistent with the unsuccessful attempts so far reported in the literature to isolate this pathogen from the vaginal cavity [60].

For example, Siavoshi et al. (2013) [30] reported the presence of *H. pylori* in vaginal yeasts by optical microscopy and amplified its genes, confirming that this bacterium is capable of harboring within yeasts [30]. Thus, it can be postulated that *H. pylori* may not reside extracellularly in the vagina and that it requires to harbor itself within yeast cells to remain viable in the vaginal environment.

The genotypes of *H. pylori* isolated in the present study, based on the amplification of virulence genes, varied. It must be emphasized that, among the bacterial profiles found in our vaginal discharge samples, a high percentage (32%) corresponded to the genotype *cagA+*, *vacAs1a/m1*, *dupA−*, which, in accordance with the literature, is associated to cases of gastric cancer [32,33,61]. It was also detected that the amplification of *vacA* gene regions provided results not described in previous studies [31,33] including strains which did not amplified any of the alleles codifying the medial region (*m1 y m2*), while others did not amplify the signal region (*s1a*, *s1b* or *s2*). To the best of our knowledge, there is no scientific literature explaining the causes which can influence this phenomenon in *H. pylori*. However, it has been observed that *Legionella pneumophila,* a bacterium pathogenic for humans which invades free living amoebas, is capable of modifying its genetic material after gaining access into the protozoa [62]. This may also be the case when *H. pylori* enters into yeasts. It has been reported that the *vacA* gene shows polymorphism in its medial, intermediate and signal regions, which vary depending on *H. pylori* strains [63] and that a defect in one of these regions may be an impediment for the bacterial strain to induce vacuolization in the infected cell [33,62,63,64]. In this sense, it is worthy to emphasize that the VacA protein plays an important role in the survival of *H. pylori* in human gastric cells because it alters endosomal traffic and promotes the accumulation of non-functional lysosomes and autophagosomes avoiding the elimination of this pathogen [65,66,67,68]. Since further knowledge on this subject is still necessary, further research will allow us to ascertain the functionality of the VacA protein variations present in the strains isolated in this study.

The phenotypic and biochemical identification of yeasts of vaginal origin harboring intracellular *H. pylori* in term pregnant women showed that 91% of the yeast-positive discharge samples contained *C. albicans*. The other two yeast species isolated also belonged to the *Candida* genus, being *C. glabrata* (7%) and *C. tropicalis (2*%). These results are comparable to those reported by Siavoshi et al., (2013) [30], who indicated that *C. albicans* was the vaginal yeast in which intracellular *H. pylori* was most frequently found (80%) but they did not identify *Candida* not *albicans* [30]. These results, altogether with the report of Sanchez-Alonzo et al. (2020) [33], in which it was demonstrated that *H. pylori* under pH stressing conditions harbors within *C. albicans*, support the hypothesis that yeasts belonging to the genus *Candida* can be included among the yeasts capable of protecting or serving as a vehicle for *H. pylori* [33].

It has been proposed that the vacuole of yeasts provides elements useful as nutrients for *H. pylori*, a proposal presently supported by numerous studies showing that *H. pylori*, as well as other bacteria, may remain viable within the vacuole of yeasts belonging to various genera [69,70,71]. Moreover, it was recently shown that stressing factors, such as inanition, may cause the exit of viable bacteria of the *Staphylococcus* genus from yeasts [72].

## 5. Conclusions

From the results of this study and that of Siavoshi et al. [30] associated to those of Matamala-Valdés [30], showing the presence of *H. pylori* within yeast cells isolated from the oral cavity of newborns, it can be suggested that *H. pylori* can, in fact, invade vaginal yeasts and allow the vertical transmission of this pathogen during birth.

## Figures and Tables

**Table 1:** Primers used for detecting and genotyping *H. pylori* contained within *Candida* yeasts.

Gene	Region	Sequence	Tm °C	Base Pairs (amplicon)	Reference
*16S rRNA*		F-5′CTCGAGAGACTAAGCCCTCC-3′ R-5′ATTACTGACGCTGATGTGC-3′	53	110	[5]
*cagA*		F-5′GATAACAGGCAAGCTTTTGAGG-3′ R-5′CTGCAAAAGATTGTTTGGCAGA-3′	55	349	[32]
*vacA*	*s1a*	F-5′-GTCAGCATCACACCGCAA-3′ R-5′-CTGCTTGAATGCGCCAAAC-3′	55	190	[33]
*vacA*	*s1b*	F-5′AGCGCCATACCGCAAGAG-3′ R-5′-CTGCTTGAATGCGCCAAAC-3′	55	187	[33]
*vacA*	s2	F-5′-GCTAACACGCCAAATGATCC-3′ R-5′-CTGCTTGAATGCGCCAAAC-3′	55	199	[33]
*vacA*	m1	F-5′-GGTCAAAATGCGGTCATGG-3′ R-5′-CCATTGGTACCTGTAGAAAC-3′	50	290	[33]
*vacA*	m2	F-5′-GGAGCCCCAGGAAACATTG-3′ R-5′-CATAACTAGCGCCTTGCAC-3′	55	352	[33]
*dupA*		F-5′-ACAAGGACGATTGAGCGATGG-3′ R-5′-TGGCTAGTTTGAGGTCTTAGG-3′	61	515	[5]

**Table 2:** Distribution and percentages of term pregnant women positive for vaginal intra-yeast *H. pylori* according to age ranges.

	Negative for Intrayeast *H. pylori*	Positive for Intrayeast *H. pylori*
Age (years)	%	%
14–20	88%	12%
21–27	85%	15%
28–34	70%	30%
35–44	67%	33%

No significant difference was observed between the positivity for vaginal intrayeast *H. pylori* and the age ranges of term pregnant women n (*p =* 0.2306).

**Table 3:** Number of samples positive for yeasts in each one of the three Family Health Centers and percentage of positive samples from the total number of positive samples.

Family Health Center	Number of Vaginal Discharge Samples	Percentage of Samples Positive for Presence of Yeasts
O’Higgins	24	14% (6/44)
Tucapel	16	11% (5/44)
Dr. Víctor Manuel Fernández	62	75% (33/44)
Total	102	100%

**Table 4:** Distribution and percentage of term pregnant women positive for yeasts harboring *H. pylori* cells according to sexual practices.

		Women Negative for Intrayeast *H. pylori*		Women Positive for Intrayeast *H. pylori*		
Sexual Practice	Answer	*n*	%	*n*	%	*p* Value
Anal sex	No	73	78%	21	22%	0.5159
	Yes	7	88%	1	13%	
Oral sex	No	68	77%	20	23%	0.4757
	Yes	12	86%	2	14%	

*n* corresponds to the number of women and % corresponds to the percentage of women for each answer.

**Table 5:** Frequency and percentage of genotypes of vaginal intrayeast *H. pylori* cells according to the amplification of the virulence genes *cagA, vacA* and *dupA.*

Genotype	Frequency	Percentage (%)
*cagA+*, *vacAs1a/m1*, *dupA−*	7	32
*cagA+*, *vacAs1a*, *dupA−*	5	23
*cagA+-*, *vacA−*, *dupA−*	1	5
*cagA+*, *vacAm1*, *dupA−*	1	5
*cagA−*, *vacAs1a*, *dupA−*	8	35
Total	22	100
